# Critical Rationalism and Trust in Science

**DOI:** 10.1007/s11191-021-00317-9

**Published:** 2021-12-20

**Authors:** Adam Chmielewski

**Affiliations:** Institute of Philosophy, University of Wrocław, Wrocław, Poland

## Abstract

In this paper, I consider whether the critical rationalist philosophy of science may provide a rationale for trusting scientific knowledge. In the first part, I refer to several insights of Karl Popper’s social and political philosophy in order to see whether they may be of help in offsetting the distrust of science spawned by the COVID-19 pandemic. In the second part, I address the more general issue of whether the theoretical principles of the critical rationalist philosophy of science may afford a foundation for building trust in science. Both parts of the discussion, confined for the sake of the argument largely to the repudiation of the concept of good reasons for considering a theory to be true, imply that this question would have to be answered negatively. Against this, I argue that such a conclusion is based on a misconception of the nature of scientific knowledge: critical rationalism views science as a cognitive regime which calls for bold theories and at the same time demands a rigorous and continuous distrust towards them, and it is precisely this attitude that should be adopted as a compelling argument for trusting science.

## Introduction: Pandemic and Science Detractors

The pandemic caused by the virus SARS-CoV-2 presents a serious challenge for science. The challenge is twofold. On the one hand, it assumes the form of conspiracy theories which undermine public trust in science by asserting that its power is purposely abused to the detriment of the people. The other part of the challenge is outright scepticism towards science. Thus far encountered mainly in the form of the anti-vaxxers movement, climate change denial, and intelligent design theories, lurking in the recesses of societies and voiced in social media, scepticism towards science seems to have received a considerable boost from the pandemic. Science detractors express their disbelief in the existence of the virus, rebel against the distress of the lockdowns imposed by most governments, spread baseless opinions about the vaccines, and promote scientifically untested bogus therapies (Cross, 2021). The ubiquity of such views suggests that science needs defending both from those who deny its worth and from those who attribute to it Mephistophelean powers which it does not have.

The notoriety of such views and attitudes in the public sphere seems to owe more to their being disproportionately amplified by the mainstream and social media, which spawn an unprecedented infodemic of false and harmful views (WHO et al., 2020), rather than to a genuine and widespread repudiation of science or to a “death of expertise” (Cassam, 2019). Though outspoken, the negation of science still appears to be a rather marginal phenomenon; it constitutes a challenge properly to be addressed by social theory and psychology and to be dealt with in the long term by systematic educational policies stressing the role of science in discovering adequate knowledge about the world. Potentially, however, it may become more widespread and dangerous than it already is. People who refuse to be vaccinated constitute a serious danger not only to themselves but also to the population at large, while people who persuade others not to be vaccinated constitute no less serious danger: misinformation kills people (WHO et al., 2020). That is why an explanation of the success of science, and building up the public trust it deserves, is both vital and urgent.

One may begin by acknowledging that the pandemic is a great opportunity not only for the detractors of science, but also for those who champion it. Scientists take a well-deserved and hard-won pride in developing numerous vaccines within just a year from the outbreak of the disease, and even greater pride in the fact that pioneering technologies were employed in their composition. The authority of epidemiological science is respected by most governments, which conform to its recommendations concerning the need for social constraints aimed at containing the virus. In the understandable hope that they will eventually be able to emerge from the paralyzing lockdowns, an overwhelming majority of people across the globe demonstrate their trust in science by obeying the rules of social distancing and by vying to be vaccinated as soon as possible. Moreover, people’s concerns about the pandemic are publicly expressed and debated with scientists, which contributes to the public awareness of the importance and value of science.

The negative impact of the COVID-19 pandemic on all spheres of human life remains in the centre of public attention. The loss of life, along with other multiple damages effected globally by the disease, as well as by attempts to control its consequences, though yet to be assessed, will certainly be momentous. The pandemic has also severely disrupted educational institutions across the globe, which is likely to contribute to a further decline in trust in science in the future. According to the United Nations, at the outset of the pandemic, 1.5 billion students suffered from the closure of their schools, from primary through secondary to tertiary levels (United Nations, 2020, p. 7). In particular, the lockdowns of educational institutions negatively affected underprivileged groups of people and whole regions, especially in the so-called Global South (Kundu & Chan et al., 2022; Mgutshini et al., 2021; Ngalim, 2021), though the negative effects of the pandemic were recorded in the countries of Global North as well. For example, American students on average suffered a variety of learning losses, but students with low socio-economic status (SES) suffered disproportionately larger losses, in part due to their lack of access to the technology required for distance learning (National Science Board, 2021, p. 8; Sanders, 2021). Similar findings were recorded in other countries (e.g. Chadwick & McLoughlin, 2021; Zagalaz-Sánchez et al., 2021). The switch to online teaching has been “not only a massive shock to children’s social life and learning pattern but also to parents” (Ngalim, 2021, p. 38). The depersonalization of the traditional relationships between pupils and teachers, which limited the conventionally available modes of motivating the pupils and focusing their attention, was one of the factors responsible for the rather moderate success of online teaching so far. The consequences of the COVID-19 pandemic “have a negative impact on the acquisition of cognitive and non-cognitive skills, affecting students’ academic performance, emotional well-being and motivation” (Ledertoug et al., 2021, p. 53), as well as imply physical impairments for the younger pupils (Zagalaz-Sánchez et al., 2021).

What is much less studied, and what opens an interesting area of exploration, is the extent to which the pandemic, along with the actions aimed at its overcoming, has also become an unprecedented public educational experience on a global scale. For example, a significant number of people in all walks of life, in their search for ways of avoiding exposure to the virus and the consequences of infection, turn not only to common-sense wisdom, otherworldly explanations, or conspiratorial theories, but also demonstrate an active and enduring interest in scientific expertise relevant to containing the disease—for instance, medicine, epidemiology, and mathematical modelling of the proliferation of the virus, in this way expanding their scientific competences (Levrini et al., 2021, p. 23; Gu & Feng, 2021).

Moreover, a number of recently published studies demonstrate that the closures have become not only an insurmountable obstacle in education, or a challenge difficult to overcome, but also an important transformative opportunity for the institutions themselves, their workers, and their students (Burgos et al., 2021; Chan et al., 2022, White & McCallum, 2021). For example, the ideas of distance learning, robustly debated for decades, but functioning thus far with moderate success only at relatively few, mostly commercial institutions, have become, overnight, a reality in educational institutions of all levels across the globe. This would not be possible without access to science-based technologies, but also without the involvement, ingenuity, and flexibility of the teaching staff of these institutions. The lockdowns have become an incentive for teachers to master new teaching tools, employ new techniques of distance teaching, prepare new curricula, and make available materials for students in a new form (Sandipan & Sanjeeva, 2020). Similarly, thanks to this experience, numerous pupils and students had to rapidly acquire and develop the digital media literacy needed to attend online schooling. This applied even to first grade pupils in primary schools, my 6-year-old daughter among them.

Lastly, it should be borne in mind that the pandemic, though caused by a particularly infectious and dangerous virus, has turned out, at least thus far, to be less deadly than other epidemics recorded in human history. Undoubtedly, this has been due to the undeniable development in many branches of science, as well as the widespread and well-organized practical application of scientific biological, medical, epidemiological theories and mathematical modelling, enhanced by powerful computation techniques, which were unavailable at the times of the former epidemics.

The above should be read as a persuasive argument in favour of trusting science. In spite of this, there is a significant number of people who not only disregard science, but also actively contest its role in society. Responding to Sibel Erduran’s important call for papers on how history, philosophy, and sociology of science may contribute to science education at the time of the pandemic (Erduran, 2020, p. 234); in this paper, I concentrate on how the philosophy of science may provide an answer to the question of why we should trust science. My attempt at answering this question will be focused on Karl R. Popper’s contribution to the philosophy and methodology of science and is divided into two parts.

In part I, I invoke several insights appropriated from Popper’s social theory. There are a number of reasons for such a choice. The first is that some parts of his work tend to be disregarded, both in contemporary theoretical debates and in curricula. This applies in particular to Popper’s philosophical contribution to social theory, in opposition to his more lasting contribution to the philosophy of science (Reiss, 2021, p. 8).

The choice of Popper’s social conceptions is not confined to a historical interest only. For, secondly, I believe that although some of his political views, described as “cold-war liberalism” (Hacohen, 2000, p. 396), are obsolete, and his criticisms of past philosophers, especially Plato, Aristotle, and Hegel, remain highly controversial and indeed faulty (e.g. Kaufmann, 1959; Levinson, 1953), his work on the theory of social science contains a number of inspiring concepts which may help us understand some aspects of our present pandemic predicament. Below, I discuss briefly Popper’s argument on the unpredictability of the future, his criticism of the conspiracy theory of society, the idea of an abstract society, and the problem of an open society. I intend to show that they retain their explanatory value and deserve to be (re)introduced into the syllabi of contemporary courses in social theory.

Thirdly, I shall attempt to show that Popper’s radically rationalist view of science is able to provide, precisely because of its radicalism, a strong rationale for trusting science. This is dealt with in part II in which I address the more technical problem of the relationship between science and technology. The discussion of the role of scientific hypotheses in designing various technological inventions is aimed at assessing the validity of the charge that Popper’s critical rationalist philosophy of science undermines trust in science instead of bolstering it. I shall attempt to show that this charge is based upon a misconceived view of the relationship between science and technology. Both parts of the paper are meant as support for the claim that an essential part of the critical rationalist theory of science is the belief that science owes its success to a systematic sceptical attitude towards hypotheses that aspire to scientific status and that science deserves to be trusted precisely because it develops through continuous distrust in itself.

## Part I

### Science and Fallibility

Science may be defined as a systematic and methodically organized cognitive activity aimed at formulating propositions about the reality that enable us to explain, predict, and transform it. Thus understood, scientific knowledge is different from the common sense, technical, artistic, religious, and magical varieties. It is also distinct from what we call wisdom, which consists of general principles of practical conduct, especially in everyday life. It is worth remarking that a scientist, even an outstanding one, does not have to be a sage, and indeed rarely is. Also, obviously, not every sage is a scholar. But science, like any other area of human life, needs wisdom. The most important wisdom of the critical rationalist philosophy of science is the repudiation of dogmatic claims to infallibility, typical of the past uncritical attitude towards science. Dogmatic belief in science, which attributed to it the ability to deliver incontestable and incorrigible truths, has been superseded by an awareness of its irremediable fallibility. The transformation in question has been the work of many minds. Karl Popper played a particularly important role in it, for he was among the first theorists of science to stress its fallibility. Popper’s hypothetic-deductive conception of science turned the fallibility of scientific knowledge from a deficiency into a virtue: his critical rationalism was based upon a belief that science owed its success to the fact that it has become a system of learning from its own failures.

Popper was a rationalist, but his rationalism differed significantly from the comprehensively rationalist doctrines of Plato, Descartes, Spinoza, or Hegel: his was critical of reason itself. His methodology, expounded in his *Logic of Scientific Discovery* (1935/2005) and in subsequent works, enjoins us to be most critical of the theories we love most: only in this way our knowledge may grow and approximate to the truth. Knowledge grows through proposing tentative and daring solutions to problems at hand and exposing them to severe testing and criticism. Popper argued that for logical reasons, an inductive verification of a hypothesis is bound to be inconclusive. Rejecting Rudolf Carnap’s confirmation, he attempted to account for the reliability of scientific knowledge by introducing the concept of corroboration, which he understood as a report from testing to which a given hypothesis has been subjected. Though he considered the context in which scientific discoveries are made to be unimportant for the logic of science, he remarked upon the social dimension of science: the testing of a hypothesis is, to some extent at least, a social undertaking in the sense that it should be formulated as clearly as possible; it should specify the range of its potential falsifiers; its formulation should avoid stratagems immunizing it from falsifications; and it should be disseminated in order to allow other scientists to criticize it.

Many of Popper’s concepts and ideas have become a part of the philosophical and, indeed, scientific vernacular. Despite this undeniable contribution to our understanding of science, Popper’s theory suffers from a number of unresolved problems (Miller, 2014). His insistence that the empirical method demands exposing the proposed hypotheses to falsifications “in every conceivable way” (Popper, 1935/2005, p. 20), and his stress on the role of negative arguments in the choice of scientific hypotheses, such as counter-examples, refutations, falsification (Popper, 1935/2005, pp. 86–88; 1979, p. 20), were criticized as unduly disregarding the need for positive confirmations which should be seen as important in selecting the most reliable hypotheses. By introducing the concept of corroboration, he seemed to have reintroduced inductive reasoning into his declaratively anti-inductivist theory. Some of his followers and collaborators, especially John Watkins (1984) and David Miller (1994, 2006), to whom I shall refer below, in their different ways attempted to rectify this apparent contradiction. Popper’s acknowledgement of the social dimensions of science was far from sufficient. It has been supplied by proponents of alternative approaches in the philosophy of science, especially by Thomas Kuhn (1962/1996), for whom Ludwik Fleck (1979) was an important source of inspiration, by scholars of the constructivist Edinburgh School with its two chief figures, David Bloor (1976/1991) and Barry Barnes (1974), as well as Latour and Woolgar (1986), and more recently by the feminist philosophers of science, among them especially Helen E. Longino (1990), Sandra Harding (1986, 1991), and Donna Haraway (1988). However, Popper’s insights into the theory of science and the theory of social science continue to be of great service in doing away with some erroneous, though persistently held, views about science.

### The Unpredictability of the Future

Despite the fact that Popper’s intuitions in the area of social and political science were formulated nearly a century ago, they may be used to cast light on some problems generated by the pandemic. In particular, they may be of use in revealing and dispelling the mental biases which blur our understanding of the global emergency. Prominent among them is the safety bias which assumes several forms. One of them is the belief in the predictability of the future. Undoubtedly, there is a deep and widespread human need to be able to prophesy what will happen in the future. This unfounded belief is dictated to us by a desire for safety: we want to know the future in order to be able to prepare ourselves beforehand for the coming events. The pandemic made us realize our own vulnerability not only by endangering each of us individually, but also by exposing the fragility of our institutions and whole societies. The future seems now more uncertain than before. Popper’s argument on the unpredictability of the future (1957; 1979) helps us deal with this uncomfortable thought precisely because it was not aimed at bringing comfort. For it makes one realize something that, as ordering-and-order-seeking beings (Chmielewski, 2020a), we tend to disregard, namely, the contingency of our condition: our lives have always been unpredictable and uncertain. Doubtlessly, in today’s rapidly transforming world, we have grown accustomed to the idea of the contingency of our individual and collective lives. Despite that, or precisely because of it, the hope of finding a niche of stability for ourselves, which would protect us from unwelcome change, is an ineradicable human craving. “All that there is is the flux of experience, bewildering and incoherent as it presents itself to us, and there is our yearning to give it order and thus to be able to enjoy it, to no longer be afraid of and threatened by it” (Jarvie, 1986, p. 234).

The aim of Popper’s argument was not so much to stress the idea of contingency but rather to make us aware that our knowledge of the world, and the actions that follow from it, significantly contribute to the contingency of the world we live in. He intended to undermine the belief in the human ability to forecast, scientifically or otherwise, the course of future events by arguing that the future cannot be predicted for logical reasons: the course of human history is largely shaped, among other factors, by the growth of our own knowledge (Popper, 1957, pp. v–vii). Yet the future growth of our knowledge cannot be predicted by any rational or scientific method. For if we were to know now what we would know in the future, it would be tantamount to us now knowing what we are supposed to know only in the future. If this were so, future knowledge would be known to us now, which is impossible. Therefore, it is not possible to predict the course of human history. Popper’s argument thus undermines the belief that science, unquestionably successful in mastering some aspects of the natural world, may also help us to master the future. Moreover, he stressed that the very desire to prevent the predicted events from happening may actually precipitate their occurrence. Such self-fulfilling prophecies, known since the times of Sophocles, he aptly called “the Oedipus effect” (Popper, 1957, p. 13). In this context it is worth remarking that Popper’s argument, though valid, does not, nor should, discourage social theorists from developing conjectural theories as to the future on the basis of empirical and historical case studies. As Bloor pointed out, since social life depends on regularity and order, some progress in establishing it will be possible (Bloor, 1991, p. 20).

Though little consolation can be drawn from this argument, in the context of the pandemic, it may be employed in two ways. One of them is to stress the Humean lesson that the belief that the future will be similar to the past is unfounded (Popper, 1979, pp. 1–31). Popper’s argument enables one to point out, for example, that shortly before the outbreak of the pandemic, even at the beginning of the year 2020, no one predicted, nor could have predicted, the appearance of the virus, the course that the disease would follow, still less the subsequent change inflicted by its spread.

The disruption of people’s daily routines by the pandemic provokes numerous calls for the restoration of normality. During the pandemic, the concept of normality grew in importance both in terms of the psychological ways of dealing with the challenges of the lockdowns, and in the normative valuation of the pre-pandemic, pandemic, as well as the expected post-pandemic reality (Levrini et al., 2021, pp. 7–8). Popper’s argument against the predictability of the future may be employed to undermine the concept of normality, which is yet another form of safety bias. It disturbingly suggests that the suspended normality we used to know until quite recently may not return, ever. Indeed, it is an oft-repeated view that from now on, nothing will be the way it used to be. Notwithstanding, we seem unable to conceive of the future as radically different from the past even though it is likely that it *will *be different. Our belief in the future being roughly the same as the past aligns with the hope that when the pandemic ends, we will not have to do anything extraordinary for the former normality to come back: after the interruption, we will just return to our regular routines and our lives will resume their previous form. This provokes also a normative question: do we, and should we, want to bring back the now-suspended normality? These questions, in turn, reveal a conservative bias in the psychological constitution of humans: it seems that now we want nothing more than a return to the normality we once knew.

Popper’s criticism of the predictability of the future does not dispel the uncertainties which presently surround us; quite the opposite, it amplifies them even more. It also suggests that a more proper attitude towards the uncertain future is to prepare oneself mentally for unexpected developments rather than to cling to an unfounded belief in the stability and predictability of the world.

### Abstract Society

Popper’s idea of the abstractness of society not only helps to portray the way of life of an increasing number of citizens of contemporary cities, but also enables one to realize how the pandemic has affected our social life. According to Popper, an abstract society is a society in which men practically never meet face to face. They do their business in isolation, communicate with each other by “typed letters or by telegrams” (Popper, 1945/1994, p. 166), and move around in closed vehicles. Even biological reproduction, supplanted by artificial insemination, would not require entering into intimate relationships. He called such a society a completely abstract or depersonalized society, and observed that in many aspects modern societies resemble this abstract model. Though we do not always drive alone in closed motor cars but meet face to face thousands of men walking past us in the street, the result is very nearly the same as if we did: we tend to avoid establishing any personal relation with our fellow pedestrians.

It is worth stressing that Popper formulated this idea in 1945, that is, at a time when many inventions which now significantly contribute to the asocial abstractness of our lives could not be known to him, namely, the Internet, e-mails, in vitro fertilization, online shopping and education, or cybersex. Nowadays, many people are detached from each other the way he described. More than half of the human population live in urbanized areas in housing facilities in which they are conveniently separated even from their immediate neighbours. In the Western world, traditional three-generational households and close-knit neighbourhoods are largely a thing of the past. The ubiquity of technology enables people to limit spontaneous personal meetings to an unavoidable or necessary minimum. An increasing number of human exchanges takes place via interfaces which mediate and replace personal contacts. People attend to their daily matters using computers, phones, and faxes and order food, clothing, entertainment, and other services online. Social media have created a new space into which people increasingly move their lives, while the traditional social, public, and intimate spheres gradually shrink. They immerse themselves in social media, where their cyber acquaintances, familiar to them as conveniently disembodied avatars, tend to receive more of their attention than tangible people within their physical reach.

Though a staunch advocate of individualism, Popper criticized the abstractness of life in modern societies by saying that many people have no or extremely few intimate personal contacts, living in anonymity and isolation, and, consequently, in unhappiness. For although society, certainly the urban one, as distinct from the rural communities, has become abstract, “the biological make-up of man has not changed much; men have social needs which they cannot satisfy in an abstract society” (Popper, 1945/1994, p. 166). In other words, contemporary societies increasingly resemble flash communities, such as those consisting of a number of people who due to sheer coincidence find themselves travelling on the same plane. Such short-lived communities are united only by the proximity imposed on them by the logic of transfer, the structure of the vehicle, and the coincidence of destination, rather than by a common origin or interest, and they disperse as soon as the plane lands in its terminus.

The pandemic has made conspicuous not only the human safety bias, but also the strength of the proximity bias. Due to a spatial or proximity preference ingrained in our minds, we value much more the familiar and the known than the distant and the foreign. Peter Singer discussed the moral dimension of the proximity bias by arguing that the moral concern we owe to a baby drowning in a pond of misery in an African slum is identical to the one which we instinctively extend to a baby drowning in our own swimming pool (Singer, 2009, p. 3), stressing that we consistently fail to observe this moral imperative. The West failed morally in the past in relation to the European Jews and fails again in relation to the refugees expelled from their homes as a consequence of Western states’ policies. The pandemic has made the proximity bias only more apparent. Understandably preoccupied with safeguarding ourselves and our nearest, we do not pay equal attention to the effects the virus will have, or already has, on the lives of people in more distant regions of the world. This lack of regard for the well-being of others is also demonstrated in the desire of developed nations to seek booster vaccinations before supporting the provision of vaccines to poorer nations unable to afford them. We care about the safety of ourselves and our loved ones, and we steer away from others as much as we can, perceiving them as a source of mortal danger. The strength of the public agoraphobia may be judged by the relief one feels when being able to invoke the constraints of social distancing in order to avoid the increasingly rare social engagements. Agoraphobic attitudes, already widespread before the pandemic, have become even more prevalent and even stronger. The lockdowns imposed on nearly all societies as a response to the pandemic, with the global resort to remote jobs and online education, only intensified the abstractness of contemporary societies. It is difficult to imagine possible ways of overcoming them in the future, and few alternative modes of cultivating sociability are forthcoming (Chmielewski, 2020b).

### Open Society Under Lockdown

The pandemic is also a time of a political lockdown of open societies. Due to the invisible danger, we are now limited also in our freedoms. This is an opportunity to realize the importance of the social relations we have been deprived of, even if they were only superficial. Far from constraining our freedom, they are its necessary precondition. It seems that the extent to which we shall be able to restore our trust in others will also reflect our ability to enjoy our freedom when the pandemic is over. Meanwhile, faced with invisible yet mortal danger, we put aside the slogans about the indispensability of freedom and docilely acquiesce in the internment imposed by the political authorities. We believe that through surrendering to the instituted constraints we will increase the chances of prolonging our lives. This reveals that the confinement is not only imposed, but also self-imposed, thus demonstrating the workings of the natural and understandable safety bias in our minds. This has an obvious political dimension.

Popper advocated an open society, as distinct from a closed one. He defined it as a society in which individuals are able to take, and are responsible for, personal decisions. In opposition to this, a closed society can be compared to an organism; it resembles a herd or a tribe—a quasi-organic unit whose members are held together by semi-biological ties. In his diagnosis of the origin of totalitarianism, which he held responsible for the closure of societies at the beginning of the twentieth century, Popper analysed the doctrine which identifies might with right (Popper, 1945/1994, p. 101). He criticized the belief according to which the very strength of a man morally legitimizes his actions, whatever their nature. Such notions were expounded by the protagonists of Plato’s dialogues, among them Thrasymachus in *The Republic*, as well as, more radically, Callicles and Polos in *The Sophist*. Against such views, Popper advocated the egalitarian philosophy of Sophists such as Antiphon, Lycophron, Hippias, and Alcidamas.

The faith in a strong political leader who, by replacing the protracted procedures of democracy, will be able to solve the fast-accumulating problems of our countries and their inhabitants is one of the most dangerous myths of contemporary social life. The myth resurfaces with renewed strength in times of uncertainty, as the present one, for it is professed nowadays by disturbingly growing masses of people, and endangers the openness of our societies. The belief in a strong man is dangerous for three reasons. First, it provokes certain individuals to present themselves as embodying the promise inscribed in such a myth. Some of them are successful in persuading people to perceive them as their earthly protectors and deliverers. In exercising the power entrusted to them by the hopeful masses, however, such individuals tend to exploit state resources to cultivate such a faith even further, thereby strengthening their power even more and immunizing themselves against democratic control mechanisms.

The second danger comes from the fact that entrusting the responsibility for the well-being of a community to an allegedly strong man undermines the sense of the responsibility of the citizens both for their own fate and for the common good of their community. These two dangers are intertwined and are equally harmful. The consequences of the faith in a strong man are deeply antidemocratic: it not only weakens individual and collective agency but also makes it difficult to depose such a strong man without resorting to violent means. As a result, a society which has relinquished its agency to a strong man will find it difficult to mobilize itself to unseat him when he does not deliver on his promises.

The third danger associated with this myth is that it overlooks and helps to eschew the educational aspects and benefits offered by participation in the democratic governance of societies on all levels. In other words, a society believing in a strong man ceases to be a genuine society and becomes an assembly of dependent and vulnerable human individuals. In view of the revived authoritarian tendencies in a number of countries, Popper’s political philosophy, which in the past was of invaluable service in dispelling the illusory benefits of delegating politics to a strong man, regains its pertinence once again.

### Pandemic as a Conspiracy

The COVID-19 pandemic is seriously aggravated by an infodemic of conspiracy theories. They explain the origin of the virus and its proliferation by outlandish, unconfirmed, distorted, and malevolent conceptions. Some of the conspiracy theorists claim that the virus was produced in a Wuhan laboratory (“the Chinese virus”) with malicious intentions; they are suspicious of the inability of science to come up with an efficient cure and proliferate eccentric theories about vaccines. Single-issue conspiracy theories, peddled prior to the emergency caused by COVID-19, effortlessly merged with new ones into allegations that the virus had been purposely produced in China for the purposes of warfare, was imported from there to control populations, and is somehow an offshoot of genetically modified organisms (GMOs). According to disseminated claims, the virus is being propagated by 5G, or chemtrails, in the interest of the so-called Big Pharma working in collusion with the political elites, and aiming, through lockdowns, to keep people in poverty, and to deprive them, through the imposition of the sanitary regime, of their freedoms. The anti-vaxxers movement, which, against the overwhelming amount of scientific data, spreads the belief in the supposed danger of vaccines, received a significant boost from the global vaccination drive to inoculate populations against COVID-19. Such conspiracy theories easily amalgamate with anti-systemic political movements like QAnon, Deep State, and similar ones, culminating in absurdities asserting, for example, that the virus was manufactured by George Soros, whereas Bill Gates intends, by means of vaccination, to microchip people in order to seize global power and to establish the One World Government. Such theories not only undermine the science-based preventive and sanative actions undertaken by the medical and political authorities. Most especially, the propagation of false information about the vaccines is a source of a grave danger to millions of people. Moreover, thanks to the widespread access to social media, such theories propagate even faster than the virus itself, which constitutes a serious challenge to the efficacy of science education.

The staggering number of false views and news about alleged conspiracies supposedly responsible for the present contagion has already become a subject of extensive scholarly studies (e.g. Bodner et al., 2021; Fuchs, 2021), as well as popular ones (e.g. Lewandowsky & Cook, 2020; Meek, 2020). They all uniformly stress that the greatest problem of the conspiratorial views is the fact that it is nearly impossible to convince the conspiracy believers that they are wrong (e.g. Bodner et al., 2021, p. 18; Lewandowsky & Cook, 2020, p. 20; Meek, 2020). Few of them, if any, refer to Popper’s work, despite the fact that he was among the first to diagnose, analyse, and criticize the phenomenon in question, which he called “the conspiracy theories of society” (Popper, 1945/1994, 1963; but see also Pigden 2006). It is evident that conspiracy theories, however bizarre, cannot be disregarded on a practical level as a marginal and innocuous phenomenon. Popper demonstrated that they are no less important from a theoretical point of view. He showed their significance for the methodology of the social sciences, and his solution to the problem they pose became part and parcel of his social theory. Though unlikely to convince any conspiracy believers that they are wrong, Popper’s approach to the problem persuasively demonstrates what is wrong with the conspiratorial way of thinking as such, and for this reason, it may be used in science education in order to inoculate people against accepting them.

In his diagnosis of the origin and structure of the conspiracy theories of society, Popper stressed that they are based upon a belief that to explain a social phenomenon is to discover the men or the group interested in the occurrence of a phenomenon which had been planned by them, and who conspired to bring it about. Such theories assert that whatever happens in society, must be a result of a design by some powerful individuals and groups (Popper, 1945/1994, p. 306). This applies especially to the events of great significance. He argued that the conspiracy theories of society are closely connected with historicism, that is, the belief in historical laws governing society, coupled with a claim that knowledge of these laws enables one to transform society according to one’s intentions. According to Popper, such theories are nothing but a secularized religious superstition: the religious belief in powerful or omnipotent gods has been supplanted by a belief in powerful people, like the Learned Elders of Zion, the imperialists, or, as “Vulgar Marxists” maintained, the capitalists responsible for the suffering of the people (Popper, 1945/1994, p. 306). Popper stressed that such theories immunize themselves from evidence which makes them difficult to debunk. For example, the present attempt to discredit such theories might be undermined by pointing out that George Soros, widely known for his many conspiracies, is not only Bill Gates’s business associate (Dawkins, 2021), but was also Popper’s pupil; this would make the present writer, bent on propagating Popper’s ideas, a part of the collusion.

Against the conspiracy theories of society Popper claimed that they are untenable because they wrongly assume that all consequences of human actions are intended. He adduces a number of illustrations demonstrating that such a view is mistaken. For example, an agent entering the market in order to pursue his economic aims influences the market in a way which undermines, however slightly, his expected results (cf. Popper, 1945/1994, p. 307). In this regard, his arguments bear some resemblance to those of Friedrich A. Hayek (Hayek, 1955, p. 84; 1960; Notturno, 2015; Shearmur, 1996), yet both thinkers significantly differed in their conclusions. Hayek took the problem of unintended consequences to be an argument against any intervention in economy or politics. Instead, he insisted on “submission to undesigned rules and conventions whose significance and importance we largely do not understand” (Hayek, 1960, p, 63). He claimed that those spheres should be left to the market forces and to the guidance of tradition from which alone there would emerge a spontaneous, and thus just, order. In opposition to this, Popper believed that the phenomenon of unintended or undesired consequences of human actions certainly undermines any revolutionary plans for the wholesale transformation of a society, which he called utopian engineering, but he wholeheartedly supported a piecemeal approach to social change. As he argued, the utopian engineer, involved in a wholesale transformation of a society, would be unable to “disentangle causes and effects, and to know what he is really doing” (Popper, 1963, p. 67), and thus could not rectify his errors. In opposition to this, the piecemeal engineer tries to implement his limited reforms “by small adjustments and re-adjustments which can be continually improved upon” (Popper, 1963, p. 66), and will have a greater chance of effecting the desired change. Moreover, he claimed that the consequences of unplanned actions may not only be unjust, but also positively harmful and will have to be remedied by a purposeful action, something which was staunchly opposed by Hayek (Notturno, 2015, p. 11).

Against conspiracy theories, Popper claimed that the main task of the theoretical social sciences is not to reveal the human intentions behind social phenomena, but to discover the unintended consequences of the intended human actions (Popper, 1963, p. 342). Against individualist psychologism, professed, among others, by J. S. Mill, Popper argued that social phenomena are to be explained not so much by “general laws” of human nature or human intentions alone, but rather by the non-psychological features of the logic of the situation in which they occur and that social situations cannot be explained by reference to motives alone (Popper, 1945/1994, p. 308). He also insisted that the method of situational logic in the social sciences is not based on “any psychological assumption concerning the rationality (or otherwise) of ‘human nature’” (Popper, 1945/1994, pp. 308–309).

To sum up, while the conspiracy theories of society are necessarily based upon an untenable belief in the efficacy of human actions, a truly scientific approach in the social sciences is to be ultimately grounded in the sceptical view that such actions will always have unpredictable and undesired consequences, whose discovery is the proper task of the theoretical social sciences (Popper, 1963, p. 342).

## Part II

### The Reliability of Science

The above might be read as an argument that Popper’s critical rationalist thought in social and political philosophy is more adept at dispelling unfounded hopes than in encouraging confidence: his philosophy is parrhetic, not consolatory. A question thus arises: can such an attitude provide a foundation for, or at least help to boost, trust in science?

According to an ancient though still widely held view, science deserves to be trusted because the knowledge it yields is true. Its truth is established through induction, that is, by mustering empirical observations in support of scientific theories. Against this Popper argued that scientific theories are universal propositions which cannot be empirically proven: the number of observations corroborating a theory we can hope to gather inductively is inevitably limited, thus inconclusive. But they can be shown to be false by providing contradicting evidence. Thus, the best we can hope for in science are theories which have not yet been falsified. In this way, the view of science as an embodiment of true knowledge foundered.

Some philosophers think this conclusion undermines the reliability of science. Reflecting upon Popper’s anti-inductivist view of science, L. Jonathan Cohen (1978) argued that by rejecting the idea of inductive support for theories, critical rationalism cuts itself off from technological objectives. Cohen claimed that one would not wish to entrust one’s life to a plane or a medicine made in accordance with the boldest conjectures which have hitherto resisted falsification. Rather, instead of Popperian appraisals of unfalsified informativeness, we need Baconian-style inductive appraisals of reliability: science should seek knowledge which is “structured by appropriate criteria of evidentially-based reliability” (Cohen, 1978, p. 11).

A view such as Cohen’s recurs frequently in discussions of critical rationalism and its view of technology. It appears in general discussions of the critical rationalist philosophy of science (e.g. Kuhn, 1962/1996; O’Hear, 1980; Oreskes, 2019; Lieberson, 1982) as well as in a great number of highly specialized ones. It was also dealt with, in the spirit of Popper’s solution of the problem of induction, by Joseph Agassi and Ian Jarvie (1987a), Ian Jarvie (1974), David Miller (e.g. 1987; 1994; 2006; 2014), Nimrod Bar-Am (2019), John Watkins (1984), David Botting (2014) and many others. While members of the critical rationalist school share Popper’s view that we have to be most critical of scientific theories, they differ as to whether Popper’s concept of corroboration implies induction or not. The disagreement, which is “genuine” (Salmon, 1981, p. 116), is the following:

We begin by asking how science can possibly do without induction. We are told that the aim of science is to arrive at the best explanatory theories we can find. When we ask how to tell whether one theory is better than another, we are told it depends on their comparative ability to stand up to severe testing. […] When we ask whether this mode of evaluation does not contain some inductive aspect, we are assured that […] since this evaluation is made entirely in terms of past performance, it escapes inductive contamination because it lacks predictive import. When we then ask how to select theories for purposes of rational prediction [and hence as a basis for rational action], we are told that we should prefer the theory which is “best tested” […], even though we have been explicitly assured that testing [has] no predictive import. (Salmon, 1981, p. 122)

I shall not endeavour to resolve this intricate issue. Instead, I shall attempt to show how the charge expressed by Cohen may be answered. I shall do so by considering, though only very cursorily and without going into complex technical details, the contributions of David Miller and John Watkins. Though they, too, differ in their views concerning induction and corroboration and have developed Popper’s ideas in divergent directions, they subscribe to the fallibilist imperative enjoining us to be most critical towards scientific theories. Then, I shall try to answer the question of whether such an attitude may be conducive to fostering trust in science.

### Doing Away with Good Reasons

Miller reinforces Popper’s rejection of justificationism, that is, a belief that scientific theories may be fully or partially justified by empirical evidence, by arguing that science has no use for good reasons which would justify the acceptance of a hypothesis. This is simply because good reasons do not exist.

Although there are such things as good arguments, and it is these that the rationalist strives to provide, there are no such things as good reasons; that is, sufficient reasons for accepting an hypothesis rather than rejecting it, or rejecting it rather than accepting it, or anything like that. Indeed, one of the finest feats of the use of reason has been the unmasking of what pose as good reasons as pawns in a hollow and fraudulent imposture. (Miller, 1987, p. 343)

He also argues that science has no use for reasons which provide only partial support for a hypothesis: “A sceptic is one, like Hume and Popper, who repudiates also the quest for partial justification” (Miller, 2006, p. 72). “Can we say that *e* provides a good reason for *h* if s(*h*, *e*), the support *h* receives from *e*, is high? It seems to me that the argument from ‘*e* supports *h*’ to ‘*e* is a good reason for *h*’, or even to ‘There is a good reason for *h*’ or ‘If *e* there is a good reason for *h*’, is just as obviously fallacious as the argument from ‘*e* logically implies *h*’ to ‘*e* is a conclusive reason for *h*’” (Miller, 1987, p. 349). After all, “an inconclusive reason for *h* is not a conclusive reason for *h*” (Miller, 1987, p. 351).

The above approach does not seem to solve the problem of choosing between two or more competing theories, *T*_1_, *T*_2_, …, *T*_*n*_, none of which has (yet) been refuted by evidence *e*. Such a problem creates a situation of uncertainty as to which of them might be true. Since in such a case evidence *e* is inconclusive, Watkins claims that scientists need a “good cognitive reason,” *other than empirical evidence*, that would help them make a rational decision as to which theory might be the best of the available ones. Such a reason, or a criterion, may be provided by the concept of the aim science is supposed to serve. If the purported aim is to be of any help in this quandary, it would have to be universally accepted, for if scientists pursued divergent aims, there would be no good, objective, and impersonal reason binding them to agree on preferring one theory over the others: a “nonarbitrary aim for science” will have to be “an aim to which all members of the republic of science could subscribe” (Watkins, 1984, p. 123).

Watkins’s concept of the aim of science is a development of an idea originally outlined by Popper (1979) and is defined in stages. First, Watkins analyses the Bacon-Descartes epistemological ideal which requires scientific theories to provide knowledge that (i) is unquestionably true; (ii) penetrates deep into phenomena, revealing their ultimate causes; (iii) forms a coherent system; (iv) is able to predict all possible changes in nature as a whole; and (v) is absolutely exact. Though such an ideal would occasion confidence in science, as it in fact did in the past, Watkins rejects it as utopian. Instead, he proposes a conjectural approach, which he arrives at by gradually downsizing this ideal. Its maximalist version is untenable because no theory can be proven to be certainly true. Untenable is also a more modest, progressivized aim, in which certain truth is supplanted by the probable one. We can only hope for theories which are *possibly* true. Accordingly, an optimum aim of science requires that we seek for theories which are possibly true, are ever deeper, more unified, more predictively powerful, and more exact (Watkins, 1984, p. 133). The aim thus defined does not point to the ultimate truth or to a path which would unerringly lead us to it. It can only steer our cognitive efforts along a “road with no known end” (Watkins, 1984, p. 355). Watkins concludes that his “neo-Popperian” theory of knowledge recognizes that the desire for epistemological security, though deep-seated:

is a yearning for a will-o’-the-wisp, for something we cannot have. The pretence that we can have it, if only we will lower our aim sufficiently, had to be buttressed by a series of increasingly severe constraints on scientific theorising. When we throw off that pretence, we free science from all these constraints and allow it to raise its aim high. If it has a pessimistic element, ours is also a liberating theory. (Watkins, 1984, p. 355)

The yearning for certainty is also dismissed by Watkins in the context of practical actions. His analysis demonstrates that real-life practical actions tend to be “recalcitrant”, that is, immune to exhaustive (game-theoretical) explanations. For this reason, he argues that an acknowledgement of the imperfection of human rationality is necessary: his Imperfect Rationality Principle is meant as a methodological guideline in an explanatory filling of the gap between actual decisions and actions, and their formal representations (Watkins, 1970).

The above arguments of Miller and Watkins, despite some important and irreconcilable differences between them, which I will not discuss, seem to undercut trust in science rather than encourage it. The epistemological safety bias, undermined by Miller through his repudiation of good reasons, is also weakened by Watkins, though by other means, i.e. by rejecting the idea of certain and probable truth and settling down for the modest possible one. From the above it also follows that the critical rationalist philosophy of science is focused on formulating principles of a very exacting cognitive regime (Chmielewski, 2020c), which demands that we be critical of our hypotheses and refuses to accept empirical evidence as deciding about their truth. This indeed does not seem to leave much ground for building trust in science and vindicates Cohen’s criticism of Popper’s anti-inductivist philosophy.

### Rescuing Good Reasons?

Two arguments may be invoked to challenge Miller’s criticism of good reasons. The first is that most people do use the concept of good reasons, though in a more relaxed, i.e. subjective (Bayesian) and inductivist sense, which differs from Miller’s demanding usage, and they are justified in doing so. Second, while good reasons may be dispensable in science, they are indispensable in technology and practical life: we want our theories to be as bold as possible, but we want our technologies to be as reliable as possible, and we need good reasons to ascertain that they are.

In relation to the first argument, let us consider, following Cohen, an example taken from aviation. Miller’s criticism might be read as implying that people awaiting their flight have no good reasons to believe their plane will take off and land safely: there is a possibility that it may crash. After all, planes do crash sometimes, and it is impossible to make sure that this particular one will not. In this sense, Miller is obviously right. But would it be rational for those people to refuse to board their plane on such grounds? Most air travellers, despite entertaining gloomy premonitions in the departure hall, do board their planes according to schedule. Some of them possibly dispel their fateful thoughts by building up their confidence through invoking a number of reasons. That the operation of planes has been perfected on the basis of scientific, technological and organizational expertise developed in a long process of trial and error, past crashes included. That the pilots have been well trained and tested. And, in relation to the particular plane that awaits them, that it has been properly serviced and that it has recently demonstrated its reliability. The fact that such reasons do play an important role in the daily decision making of millions of travellers, travelling scientists and philosophers among them, could be taken as a good reason to call them “good reasons”: after all, past experience of millions of people cannot be wholly deceptive. Against this, it may be said that it is quite obvious that most people seem to be consistent Humeans by simultaneously holding two inconsistent beliefs: while entertaining pessimistic thoughts, they still get on the plane, thus falling into a performative inconsistency. The reasons they recite to themselves do not provide firm grounds for predicting a safe journey. Passengers of the two Boeing 737 MAX planes which crashed in 2018 and 2019 had an additional good reason in this subjectivist and inductivist sense: their planes were brand new, state-of-the-art productions of a world leader in aviation. It thus seems that just as good reasons do not exist in science, one cannot make much use of them outside science either, and the first argument turns out to be only “displeasingly verbal” (Miller, 1987, p. 345).

### The Regime of Science

In order to deal with the second argument, let us return to the above definition of science as a cognitive activity organized into a systematic and methodical regime aimed at formulating propositions that enable us to explain, predict, and transform reality. Though borderlines between science and other types of knowledge are porous, it differs from them. Without aspiring to supplant common sense, science grows upon it and surpasses it (Popper, 1979, p. 33). The picture of reality created by science may be and often is beautiful, and its construction may involve artistic skills, but its purpose cannot be reduced to aesthetic values. Science differs from magic, which is based upon the belief in a metonymic instead of symbolic relationship between words and things, and is not about giving us only an illusion of power (I take here a different view from Agassi & Jarvie, 1987b, 1987c). The aim of scientific knowledge, as distinct from its religious counterpart, is not to bring consolation, though religion may inspire—as well as stifle—scientific insights.

Science is also different from technological knowledge: though it does play a role in technology, it pursues different aims. Science is a domain of theoretical reason which is concerned with how things are, while technology is a domain of practical reason which is concerned with how to do things. Moreover, scientific theories which strive to accurately describe things are not a sufficient condition for successfully doing things, and often not even a necessary one. Arguments like the one expressed by Cohen imply, however, that technology is some sort of a deduction from, or a spin-off of, scientific theories, which have to be inductively supported prior to their application in technology. This view, though widely shared, overlooks the fact that practical purposes may be reliably served with the help of scientific theories which have been conclusively falsified, or even without theoretical backing. The standard example is Ptolemy’s geocentric model of the universe which, though superseded by the Copernican model, is sufficient for successfully navigating at sea. Other examples abound. Humans knew how to melt metals well before they had any metallurgic theory or even learned to measure temperature. Newton’s mechanics was superseded by Einstein’s theory of relativity, yet it works well even in engineering human journeys into outer space. People learned how to fly machines before they had a theory of lift. The mathematical laws of thermodynamics were formulated only after a century since steam engines were employed in the mining industry for the first time. London pigeon fanciers knew how to cross the birds in order to endow their new generations with fancy forms without instruction from Darwin’s theory of evolution; as a matter of historical fact, it was the other way around: the knowledge Darwin acquired from the fanciers helped him to develop his theory of natural selection (“Darwin could see no substitute for hobnobbing with fanciers. It was the only way to pick up the lore” [Desmond & Moore, 1991, p. 429]). Rigorous scientific explanations may, and often are, subsequently employed in the construction of ever more sophisticated technologies than those which originally provoked their emergence, yet it is a task for technicians assisted by the applied, not the theoretical sciences (Agassi, 1974).

An investigation into the crashes of the Boeing 737 MAX planes demonstrated that they were caused by faulty software design. The investigation also revealed that sloppiness in production, unsatisfactory supervision, and corporate greed significantly contributed to the disasters. Worst of all, it turned out that the Federal Aviation Administration, responsible for certifying the plane in question, had entrusted the job to its producer, delegating in this way the public trust placed in it to the interested party, grossly disregarding the evident conflict of interest (“Excessive FAA delegation of certification functions to Boeing on the 737 MAX eroded FAA’s oversight effectiveness and the safety of the public” [The House Committee on Transportation & Infrastructure, 2020, p. 64]). No scientific theories were blamed for the accidents, though. This was so because the theories employed in the construction of the planes, prior to their employment, had been subjected to the rigours of the cognitive regime which critical rationalism strives to define. A distrustful attitude towards scientific hypotheses is crucial for eliminating the unavoidable errors of their authors because no scientist is blessed with epistemic grace (Bloor, 2007, p. 267). It is necessary also, or rather especially, because some of them think otherwise, or are biased due to their gender or political views.

Science deserves to be trusted in so far as it is based on a rigorous distrust of itself. It follows that if science is to be relied upon in the design of reliable technologies, be it a plane or a vaccine, the proper attitude is to institutionalize the demand for bold theories and a distrust of them, rather than leniency. The institutionalization of this demand lies at the foundation of the regime of science. Yet, the institutionalized regime of science is embedded in a number of other regimes. The social, cultural, and political ones, in which scientific practice is grounded, and which enable science to function in the first place, influence at the same time, sometimes adversely, the pursuit of the aim of science: their overlapping, dynamic, and constraining influences affect in various ways the production of scientific knowledge (Jasanoff, 2004).

Trust in science depends also upon the social context within which scientific theories are employed, through technology, in serving the public. This area of investigation thus far has not been of paramount importance for critical rationalists (Ormerod, 2013, p. 469). The above argument indicates that technological instruments are more likely to fail and endanger human safety not because of insufficient inductive support for scientific theories, but rather due to the disfunctions of the public institutions which are responsible for enabling these theories’ technological employment. All too often such institutions turn out to be ill-designed and badly manned (Popper, 1945/1994, p. 120), as well as improperly managed and impervious to change generated by the growth of knowledge and technology. What is thus needed is a sustained reflection on how to establish, manage, and reform the institutional, organizational and political regimes charged with the task of serving and protecting the public interest by rigorously controlling the technologies offered to the general public.

## Data Availability

Not applicable.

## References

[CR1] Agassi J, Rapp F (1974). The confusion between science and technology in the standard philosophies of science. Contributions to a philosophy of technology: Studies in the structure of thinking in the technological sciences.

[CR2] Agassi, J., & Jarvie, I. C. (1987a). *Rationality: The critical view*. Martinus Nijhoff.

[CR3] Agassi, J., & Jarvie, I. C. (1987b). The problem of the rationality of magic. In J. Agassi & I. C. Jarvie, *Rationality: The critical view* (pp. 364–383). Martinus Nijhoff.

[CR4] Agassi, J., & Jarvie, I. C. (1987c). Magic and rationality again. In J. Agassi & I. C. Jarvie, *Rationality: The critical view* (pp. 385–394). Martinus Nijhoff.

[CR5] Bar-Am, N. (2019). How should social engineers develop critical social science? In R. Sassower & N. Laor (Eds.), *The impact of critical rationalism: Expanding the Popperian legacy through the works of Ian C. Jarvie* (pp. 9–18). Palgrave MacMillan.

[CR6] Barnes B (1974). Scientific knowledge and sociological theory.

[CR7] Bloor, D. (1991). *Knowledge and social imagery* (2nd ed.). The University of Chicago Press. (Original work published 1976)

[CR8] Bloor D (2007). Epistemic grace: Antirelativism as theology in disguise. Common Knowledge.

[CR9] Bodner, J., Welch, W., Brodie, I., Muldoon, A., Leech, D., & Marshall, A. (Eds.). (2021). *COVID-19 conspiracy theories: QAnon, 5G, the New World Order and other viral ideas*. McFarland.

[CR10] Botting D (2014). Probability and Rational Choice. Principia.

[CR11] Burgos, D., Tlili A., & Tabacco, A. (Eds.). (2021). *Radical solutions for education in a crisis context: COVID-19 as an opportunity for global learning*. Springer.

[CR12] Cassam Q (2019). Conspiracy theories.

[CR13] Chan, R. Y., Bista, K., & Allen, R. M. (Eds.). (2022). *Online teaching and learning in higher education during COVID-19: International perspectives and experiences*. Routledge.

[CR14] Chmielewski A (2020). Politics and recognition: Towards a new political aesthetics. Routledge.

[CR15] Chmielewski A (2020). Abstract society in the time of plague. Philosophy of the Social Sciences.

[CR16] Chmielewski, A. (2020c). Post-truth and consequences. In M. Gudonis & B. T. Jones (Eds.), *History in a post-truth world: Theory and praxis* (pp. 46–65). Routledge. 10.4324/9780429319204-4

[CR17] Cohen, J. L. (1978, July 14). Is Popper more relevant than Bacon for scientists? *The Times Higher Education Supplement*, 10–11.

[CR18] Cross, R. (2021, January 25). Will public trust in science survive the pandemic? *Chemical and Engineering News*, *99*(3). https://cen.acs.org/policy/global-health/Will-public-trust-in-science-survive-the-pandemic/99/i3

[CR19] Dawkins, D. (2021). George Soros and Bill Gates’ backed consortium to buy U.K. maker of Covid tests for $41 million. *Forbes*. https://www.forbes.com/sites/daviddawkins/2021/07/19/george-soros-and-bill-gates-backed-consortium-to-buy-uk-maker-of-covid-lateral-flow-tests-for-41-million/?sh=743e41c42687

[CR20] Desmond, A., & Moore, J. (1991). *Darwin*. Warner Books.

[CR21] Erduran S (2020). Science education in the era of a pandemic. How can history, philosophy and sociology of science contribute to education for understanding and solving the Covid-19 crisis?. Science & Education.

[CR22] Fleck, L. (1979). *Genesis and development of a scientific fact* (F. Bradley, Trans.; T. J. Trenn & R. K. Merton, Eds.), The University of Chicago Press.

[CR23] Fuchs, Ch. (2021). *Communicating COVID-19: Everyday life, digital capitalism, and conspiracy theories in pandemic times*. Emerald.

[CR24] Gu C, Feng Y (2021). Influence of public engagement with science on scientific information literacy during the COVID-19 pandemic: Empirical evidence from college students in China. Science & Education.

[CR25] Hacohen, M. H. (2000). *Karl Popper: The formative years, 1902–1945. Politics and philosophy in interwar Vienna*. Cambridge University Press.

[CR26] Haraway D (1988). Situated knowledges: The science question in feminism and the privilege of partial perspective. Feminist Studies.

[CR27] Harding S (1986). The science question in feminism.

[CR28] Harding S (1991). Whose science? Whose knowledge?.

[CR29] Hayek, F. A. (1955). *The counter-revolution of science: Studies on the abuse of reason*. The Free Press.

[CR30] Hayek FA (1960). The constitution of liberty.

[CR31] Jarvie IC, Rapp F (1974). The social character of technological problems. Contributions to a philosophy of technology: Studies in the structure of thinking in the technological sciences.

[CR32] Jarvie, I. C. (1986). *Thinking about society: Theory and practice* (Boston Studies in the Philosophy of Science, Vol. 93). Reidel.

[CR33] Jasanoff S, Jasanoff S (2004). The idiom of coproduction. States of knowledge: The co-production of science and the social order.

[CR34] Kaufmann, W. (1959). *From Shakespeare to existentialism: Studies in poetry, religion, and philosophy*. Beacon Press.

[CR35] Kuhn, T. S. (1996). *The structure of scientific revolutions*. The University of Chicago Press. (Original work published 1962)

[CR36] Latour, B., & Woolgar, S. (1986). *Laboratory life: The construction of scientific facts*. Princeton University Press.

[CR37] Ledertoug, M. M., Tidmand, L., Las Hayas, C., Gabrielli S., & Carbone, S. (2021). Upright: Well-being & resilience education: Disrupted by the COVID-19 pandemic. In M. A. White & F. McCallum. (Eds.), *Wellbeing and resilience education: COVID-19 and its impact on education* (pp. 51–76). Routledge.

[CR38] Levinson RB (1953). In defense of Plato.

[CR39] Levrini O, Fantini P, Barelli E, Branchetti L, Satanassi S, Tasquier G (2021). The present shock and time re-appropriation in the pandemic era: Missed opportunities for science education. Science & Education.

[CR40] Lewandowsky, S., & Cook, J. (2020). *The conspiracy theory handbook.* http://sks.to/conspiracy

[CR41] Lieberson, J. (1982, November 18). The ‘truth’ of Karl Popper. *The New York Review of Books*. https://www.nybooks.com/articles/1982/11/18/the-truth-of-karl-popper/

[CR42] Longino, H. E. (1990). *Science as social knowledge: Values and objectivity in scientific inquiry*. Princeton University Press.

[CR43] Meek, J. (2020, October 22). Red pill, blue pill. *London Review of Books*, *42*(20). https://www.lrb.co.uk/the-paper/v42/n20/james-meek/red-pill-blue-pill

[CR44] Mgutshini, T., Oparinde, K., & Govender, V. (Eds.). (2021). *COVID-19: Interdisciplinary explorations of impacts on higher education*. Sun Press.

[CR45] Miller DW, Agassi J, Jarvie IC (1987). A critique of good reasons. Rationality: The critical view.

[CR46] Miller, D. W. (1994). *Critical rationalism: A restatement and a defence*. Open Court.

[CR47] Miller, D. W. (2006). *Out of error: Further essays on critical rationalism*. Ashgate.

[CR48] Miller DW (2014). Some hard questions for critical rationalism. Discusiones Filosóficas.

[CR49] National Science Board. (2021). *Science and engineering indicators*. https://ncses.nsf.gov/pubs/nsb20211/online-education-in-stem-and-impact-of-covid-19

[CR50] Ngalim, A. N. (2021). COVID-19 lockdown teaching and learning responses in Cameroon: Hurdles and opportunities. In Kundu, N. D., & Ngalim A. N. (Eds.). (2021). *COVID-19: Impact on education and beyond*. (pp 38–53). Vij Books.

[CR51] Notturno MA (2015). Hayek and Popper: On rationality, economism, and democracy.

[CR52] O’Hear A (1980). Karl Popper.

[CR53] Oreskes N (2019). Why trust science?.

[CR54] Ormerod R (2013). Logic and rationality in OR interventions: An examination in the light of the ‘critical rationalist’ approach. Journal of Operational Research Society.

[CR55] Pigden Ch, Coady D (2006). Popper revisited, or what is wrong with conspiracy theories?. Conspiracy theories: The philosophical debate.

[CR56] Popper, K. R. (1963). *Conjectures and refutations: The growth of scientific knowledge*. Routledge & Kegan Paul.

[CR57] Popper KR (1957). The poverty of historicism.

[CR58] Popper, K. R. (1979). *Objective knowledge: An evolutionary approach*. Oxford University Press.

[CR59] Popper, K. R. (1994). *The open society and its enemies*. Princeton University Press. (Original work published 1945)

[CR60] Popper, K. R. (2005). *The logic of scientific discovery*. Routledge. (Original work published 1935).

[CR61] Reiss MJ (2021). Science education in the light of COVID-19: The contribution of history, philosophy and sociology of science. Science & Education.

[CR62] Chadwick R, McLoughlin E (2021). Impact of the COVID-19 crisis on learning, teaching and facilitation of practical activities in science upon reopening of Irish schools. Irish Educational Studies.

[CR63] Salmon WC (1981). Rational prediction. British Journal for the Philosophy of Science.

[CR64] Sanders, L. (2021, August 10). *What kids lost when COVID-19 upended school*. Science News. https://www.sciencenews.org/article/covid-school-kids-lost-education-learning-gap

[CR65] Sandipan R, Sanjeeva S (2020). Virtualization of science education: A lesson from the COVID-19 pandemic. Journal of Proteins and Proteomics.

[CR66] Shearmur, J. (1996). *Hayek and after: Hayekian liberalism as a research programme*. Routledge.

[CR67] Singer, P. (2009). *The life you can save: Acting now to end the world poverty*. Random House.

[CR68] The House Committee on Transportation and Infrastructure. (2020). *Final Committee report: The design, development & certification of the Boeing 737 MAX*. https://transportation.house.gov/imo/media/doc/2020.09.15%20FINAL%20737%20MAX%20Report%20for%20Public%20Release.pdf

[CR69] United Nations. (2020, April). *The impact of COVID-19 on children: Policy brief*. https://unsdg.un.org/resources/policy-brief-impact-covid-19-children

[CR70] Watkins JWN, Borger R, Cioffi F (1970). Imperfect rationality. Explanation in the behavioural sciences.

[CR71] Watkins JWN (1984). Science and scepticism.

[CR72] White, M. A., & McCallum, F. (Eds.). (2021). *Wellbeing and resilience education: COVID-19 and its impact on education*. Routledge.

[CR73] WHO et al. (2020, September 23). *Managing the COVID-19 infodemic: Promoting healthy behaviours and mitigating the harm from misinformation and disinformation. Joint statement by WHO, UN, UNICEF, UNDP, UNESCO, UNAIDS, ITU, UN Global Pulse, and IFRC*. World Health Organization. https://www.who.int/news/item/23-09-2020-managing-the-covid-19-infodemic-promoting-healthy-behaviours-and-mitigating-the-harm-from-misinformation-and-disinformation

[CR74] Zagalaz-Sánchez ML, Cachón-Zagalaz J, Arufe-Giráldez V, Sanmiguel-Rodríguez A, González-Valero G (2021). Influence of the characteristics of the house and place of residence in the daily educational activities of children during the period of COVID-19’ confinement. Heliyon.

